# AMF/PGI-mediated tumorigenesis through MAPK-ERK signaling in endometrial carcinoma

**DOI:** 10.18632/oncotarget.4708

**Published:** 2015-07-20

**Authors:** Yiran Li, Yuanhui Jia, Qi Che, Qian Zhou, Kai Wang, Xiao-Ping Wan

**Affiliations:** ^1^ Department of Obstetrics and Gynecology, Shanghai First People's Hospital Affiliated to Shanghai Jiao Tong University, Shanghai, China; ^2^ Clinical and Translational Research Center, Shanghai First Maternity and Infant Hospital, Tongji University School of Medicine, Shanghai, China; ^3^ Department of Gynecology, Shanghai First Maternity and Infant Hospital, Tongji University School of Medicine, Shanghai, China

**Keywords:** endometrial carcinoma, Autocrine Motility Factor (AMF)/PhosphoGlucose Isomerase (PGI), MAPK signaling pathway, invasion, prognosis

## Abstract

Autocrine motility factor (AMF), which is also known as phosphoglucose isomerase (PGI), enhances tumor cell growth and motility. In this study, we found that AMF and its receptor were both highly expressed in Endometrial Carcinoma (EC) tissues compared to normal tissues. Levels of AMF were increased in serum of endometrial cancer patients. Downregulation of AMF by shRNA inhibited invasion, migration and proliferation as well as growth in a three-dimensional culture. AMF cytokine function, but not enzymatic activity of PGI, regulated tumorigenic activities of AMF. The MAPK-ERK1/2 pathway contributed to AMF-induced effects in EC cells. In agreement, Mek inhibitor decreased AMF-induced invasion, migration and proliferation of EC cells. In addition, in two mouse tumor metastasis models (EC cells delivered through left ventricle or intraperitoneally) AMF-silenced EC cells showed decreased tumor proliferative and metastatic capacities. We suggest that AMF/PGI is a potential therapeutic target in endometrial carcinoma.

## INTRODUCTION

Endometrial carcinoma (EC) is the most frequent gynecological malignancy and a leading cause of cancer death in women worldwide. In 2014, in the USA alone, approximately 6% (52,630) of total new cancer cases and 3% (8,590) of total cancer deaths were from EC. [1] Evidence indicates that although early-stage EC is often successfully treated with surgical intervention, treatments for late-stage EC are difficult and the prognosis is poor [2, 3]. In particular, EC cases with regional or distal metastasis result in a much more dismal outcome [4]. Thus, it is critical to further elucidate the molecular and cellular mechanisms underlying EC tumorigenesis and to develop novel diagnostic and prognostic biomarkers, as well as therapeutic strategies, for EC.

Cancer progression and metastasis are controlled by various extracellular growth factors and cytokines [5, 6]. One such factor, autocrine motility factor (AMF), which is also known as phosphoglucose isomerase (PGI), was originally isolated from the conditioned medium of melanoma cancer cells and stimulates both direct and random cell migration [7, 8]. However, this protein also behaves as a housekeeping cytosolic enzyme of sugar metabolism and plays a key role in both glycolysis and gluconeogenesis pathways, catalyzing the interconversion of glucose 6-phosphate and fructose 6-phosphate [9]. Before the identification of its primary sequence, it was also known by other names: neuroleukin, which promotes growth of embryonic spinal and sensory neurons [10], a maturation factor mediating differentiation of human myeloid leukemia cells [11], sperm antigen-36 [12], and a myofibril-bound serine proteinase inhibitor [13]. Several researchers independently found that secreted AMF, a tumor-secreted cytokine, is involved in regulation oncogenesis and tumor progression in various human cancers including breast [14, 15], lung [16], liver [17, 18], melanoma [19]and osteogenic sarcoma [20]. In clinical cancer pathology, the presence of AMF in serum and urine is one of the prognostic biomarkers that indicate cancer progression [21–23]. Extracellular AMF binds to its receptor, autocrine motility factor receptor, E3 ubiquitin protein ligase (AMFR), a 78-kDa seven-transmembrane glycoprotein [24, 25], and subsequently stimulates numerous signaling pathways, including those governed by protein kinase C (PKC) and tyrosine kinase, the small GTPases Rac1 and RhoA, and downstream JNK [26–28]; receptor binding also leads to the activation of phosphatidylinositol 3-kinase (PI3-kinase) and mitogen-activated protein kinase (MAPK) pathways, thereby regulating the proliferative and angiogenic activities of tumor cells [29–32]. Recent studies have indicated that AMF/PGI binds HER2, a family member of EGF receptors (HER), leading to the activation of the PI3K/AKT and MAPK/ERK pathways to enhance tumor cell motility in breast cancer [14, 15]. Currently, the specific role of AMF/PGI in human EC remains unclear, and therefore it is of considerable interest to identify novel mechanisms to better understand how EC occurs and disseminates.

In this paper, we first demonstrated that AMF/PGI is significantly correlated with EC aggressiveness from all aspects of clinical EC patients, EC cell lines and EC mouse models. First, we tested AMF expression in EC patients. Then, we examined the biological consequences of AMF downregulation in aggressive human EC cell lines. Moreover, phospho-antibody microarrays revealed that the MAPK-ERK1/2 signaling pathway is involved in AMF-mediated endometrial cancer and that the re-activation of MAPK-ERK1/2 signaling with exogenous PGI in co-cultured EC cells induced migration, invasion and proliferation. At last, we verified our finding using *in vivo* mouse models and found that the silencing of AMF abrogated tumor growth and lowered the level of MAPK-ERK1/2 signaling phosphorylation. These results shed light on the mechanisms and pathways by which EC occurs and develops, providing evidence that AMF/PGI is a novel proto-oncoprotein of EC and therefore a potential therapeutic target.

## RESULTS

### AMF is highly expressed in EC tissues and serum of EC patients

**Figure 1:**
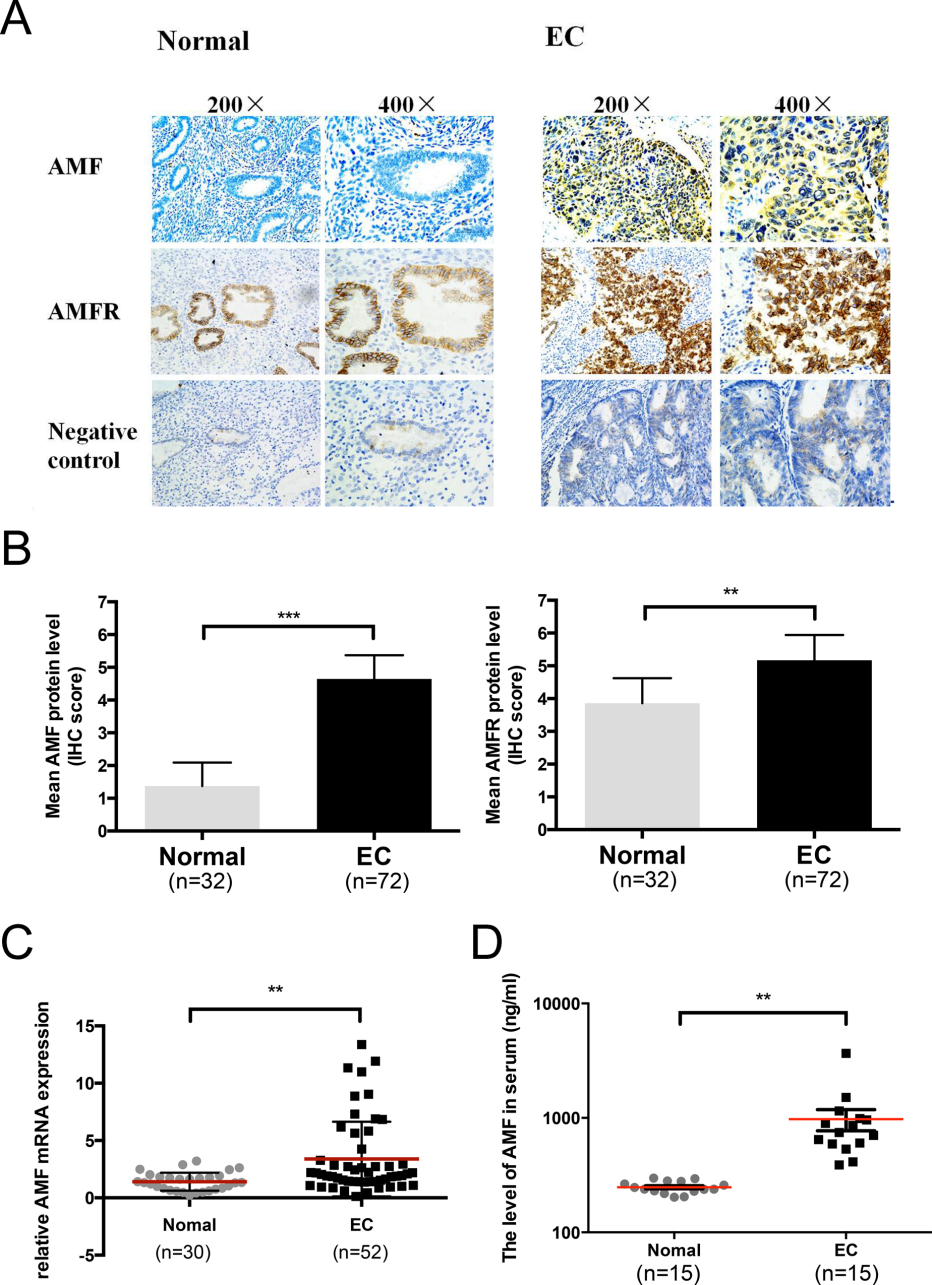
Autocrine motility factor is highly expressed in EC tissues and serum **A.** Immunohistochemical analysis of AMF and AMFR expression in normal endometrium and endometrioid cancer (EC). **B.** Summary of the IHC score in normal endometrium and EC (***p < 0.001). **C.** AMF expression in EC tissue specimens (*n* = 52) and normal endometrium (*n* = 30) was assessed by qRT-PCR and normalized to β-actin expression (**P < 0.01, unpaired Student's *t* test). **D.** The levels of AMF in serum of patients with EC (*n* = 15) and normal endometrium (*n* = 15) were measured by ELISA (**P < 0.01, unpaired Student's *t* test).

AMF and its receptor AMFR expressions were evaluated in normal endometrium (32 samples) and endometrial cancer (72 samples) tissues using immunohistochemistry (IHC). AMF and AMFR were both overexpressed in EC tissues compared with the normal endometrium. AMF was found predominantly with cytoplasmic staining and AMFR was located mainly on the cell surface. Normal tissues stained nearly negative for AMF and weakly positive for AMFR; however, tumor tissues exhibited strong staining for both AMF and AMFR. (*P* < 0.01) (Figure 1A, 1B).

Furthermore, AMF mRNA levels were quantified by qRT-PCR. AMF mRNA levels were significantly higher in the EC tissues (52 cases) than in the normal endometrium specimens (30 cases) (*P* < 0.01) (Figure 1C). We verified the AMF level in serum according to the expression of AMF in endometrium tissue. AMF concentration in serum of 15 patients with untreated endometrial cancer and 15 normal women (control group) were examined and we found that there was remarkable increase in AMF secretion in the serum of EC patients (*P* < 0.01) (Figure 1D) compared with that in control group. These data indicated that AMF expression was much higher in EC tissue and serum than in normal endometrium and normal serum.

### Effect of AMF gene silencing on EC cells migration and invasion

**Figure 2:**
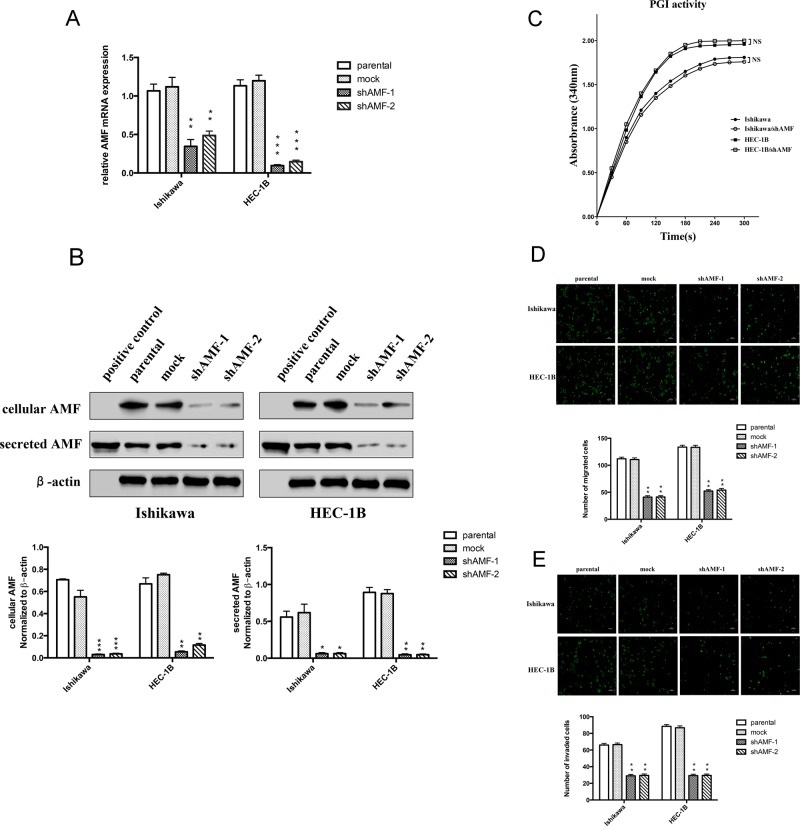
Effect of AMF gene silencing on EC cells migration and invasion **A.** Ishikawa and HEC-1B cells were stably transfected with plasmid containing AMF-specific shRNA (shAMF-1 or shAMF-2)) or control plasmid (mock). Cells were then analyzed by qRT-PCR. **B.** Top, Immunoblot analysis for AMF protein and β-actin expression; Bottom, quantification analysis of AMF expression. AMF cytokine itself was used as positive control. **C.** Intracellular PGI/AMF enzyme activity was measured in EC cell lines by enzymatic spectrophotometer assay. **D.** and **E.** Transwell assay for cells migration (D) and invasion (E) Cells were seeded onto the upper surface of Transwell chambers without (D) or with Matrigel-coated (E) After 16 h (D) or 24 h (E) of incubation, the penetrating cells were stained with calcein-AM and recorded under a microscope. Top, Photographs depict the migration or invasion of EC cells; Bottom, quantitative data are expressed as mean average ± SD from three independent experiments. (*, *P* < 0.05; **, *P* < 0.01, ***P < 0.001, compared with the control cells).

Migration and invasion are important prerequisites for tumor progression and metastasis. To determine the role of AMF silencing in EC progression, we stably transfected EC cell lines Ishikawa and HEC-1B. We chose these lines due to their highly endogenous AMF expression, confirmed by Western blot (Data is not shown). We used lentiviral vectors encoding shRNA targeted towards human AMF (Ishikawa/shAMF-1, Ishikawa/shAMF-2, HEC-1B/shAMF-1 and HEC-1B/shAMF-2) and an empty vector for a control (Ishikawa/mock and HEC-1B/mock). To examine the efficiency of AMF silencing, the levels of mRNA and protein expression were detected in the transfectants (Figure 2A and 2B); silencing of endogenous AMF by shRNA led to its near complete depletion (Figure 2B). We observed similar changes in protein expression using two target shRNA sequences against AMF (shAMF-1 and shAMF-2), suggesting that the suppression of AMF is not due to an off-target effect of the shRNA.

After silencing of AMF expression, the next obvious question was to find out whether shAMF cells exhibited decreased enzymatic activity of PGI. To address the possibility that AMF silencing inhibited intracellular PGI activity in glycolytic metabolism, we measured intracellular PGI activity and found that silencing of AMF by shRNA did not affect the enzymatic activity of PGI in both EC cell lines (Figure 2C). Next, the *in vitro* transwell assay was designed to test whether transfection of shAMF altered the locomotive potential of tumor cells. After 16 h of incubation, reduction of AMF resulted in a significant decrease in cell migration (Figure 2D). To study the effect of shAMF transfection on cell invasion, parental and transfected cells were seeded on Matrigel-coated Transwell chambers. The ability of shAMF cells to invade through Matrigel decreased dramatically compared with that of the control cells (Figure 2E). In conclusion, AMF silencing significantly suppressed the migration and invasion capabilities of Ishikawa and HEC-1B cells via reducing extracellular AMF, but not intracellular PGI/AMF.

### Effect of AMF gene silencing on EC cells proliferation, cell cycle from G0/G1 to S phase transition and spheroid-forming ability

**Figure 3:**
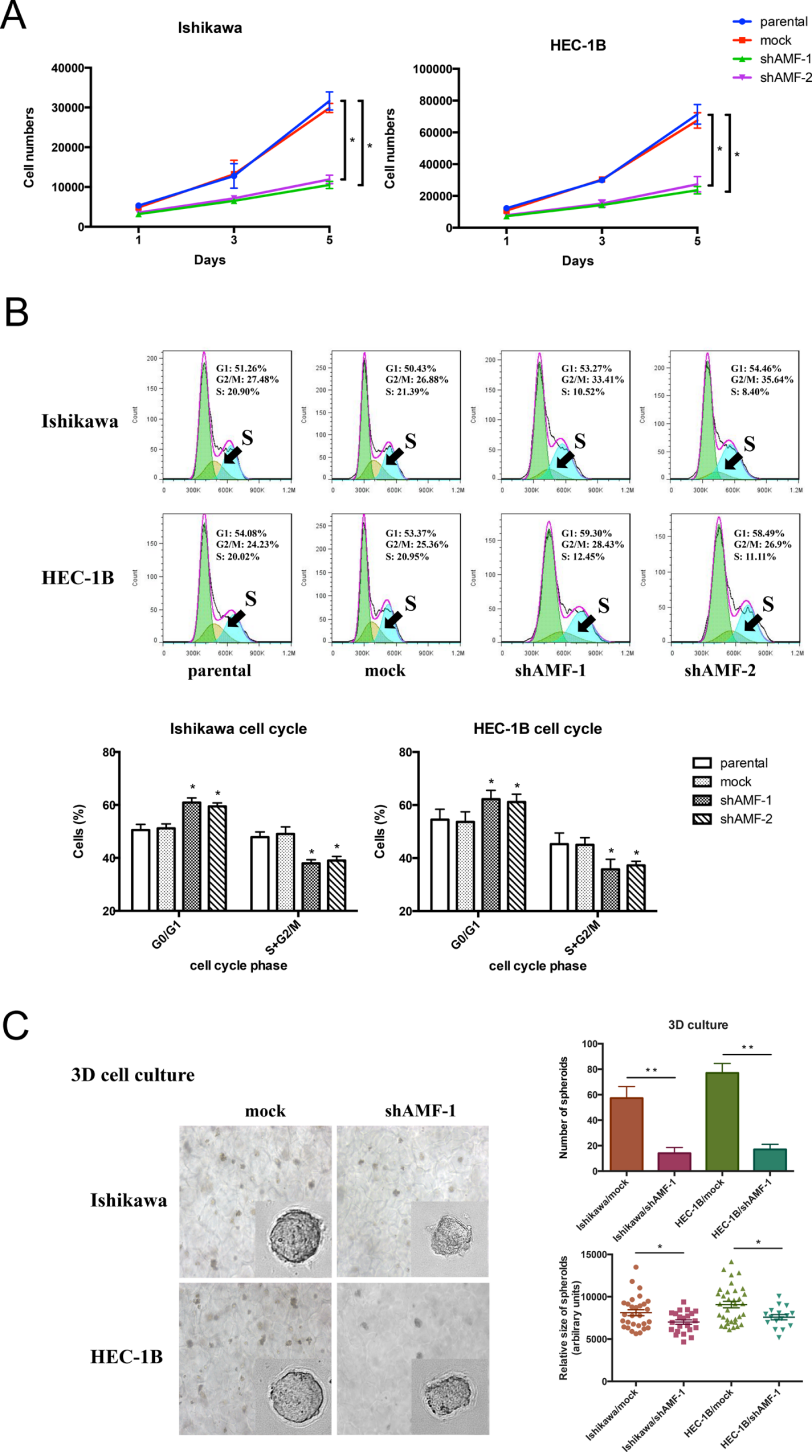
Effect of AMF gene silencing on EC cells growth **A.** Growth curve by MTS assay. Cells were seeded at low density (2000 per well) and grown for 5 d. Fresh medium was provided everyday (Points, mean of triplicate determinations; bars, SD). **B.** Cell cycle profile was analyzed by fluorescence-activated cell sorting (FACS); depletion of AMF in endometrial cells altered the cell cycle by arresting the G0/G1 to S phase transition. The summary graphs were presented below the cell cycle profiles. Data represent the mean ± S.D. of three independent experiments (*P < 0.05). **C.** Ishikawa/shAMF-1, HEC-1B/shAMF-1 and the spheroid formation of the controls in 3D culture was photographed at day 14 in culture (representative images are shown; 400× for the inserts, 200× for all others; scale bar, 100 μm). Quantification of the relative size (lower right) and number (lower left) of spheroids. (*P < 0.05; **P < 0.01)

A cell proliferation assay was used to investigate the effect of AMF silencing on cell proliferation. Silencing of the AMF gene decreased the number of viable cells, whereas control cells continued to proliferate normally. (Figure 3A).

Next, we performed cell cycle analysis to determine whether AMF knockdown inhibits cells growth via alteration of the cell cycle. Reduction of AMF expression in Ishikawa/shAMF-1, Ishikawa/shAMF-2, HEC-1B/shAMF-1 and HEC-1B/shAMF-2 inhibited the cell cycle by arresting the G0/G1-to-S phase transition compared with that in the parental and mock controls (Figure 3B), suggesting AMF functions as a cell cycle priming factor in EC cells.

To further confirm that AMF promotes tumor growth, we employed a three-dimensional spheroid culture system to mimic physiological conditions of tumor growth and compared the spheroid-forming ability of the AMF knockdown cells with that of the control cells. Consistently, both the size and number of spheroids formed was reduced in Ishikawa/shAMF-1 cells and HEC-1B/shAMF-1 cells compared with that in mock controls (Figure 3C). Taken together, these results suggest that AMF might affect EC tumor cell growth.

### MAPK signaling pathway is involved in AMF-mediated activation

**Figure 4:**
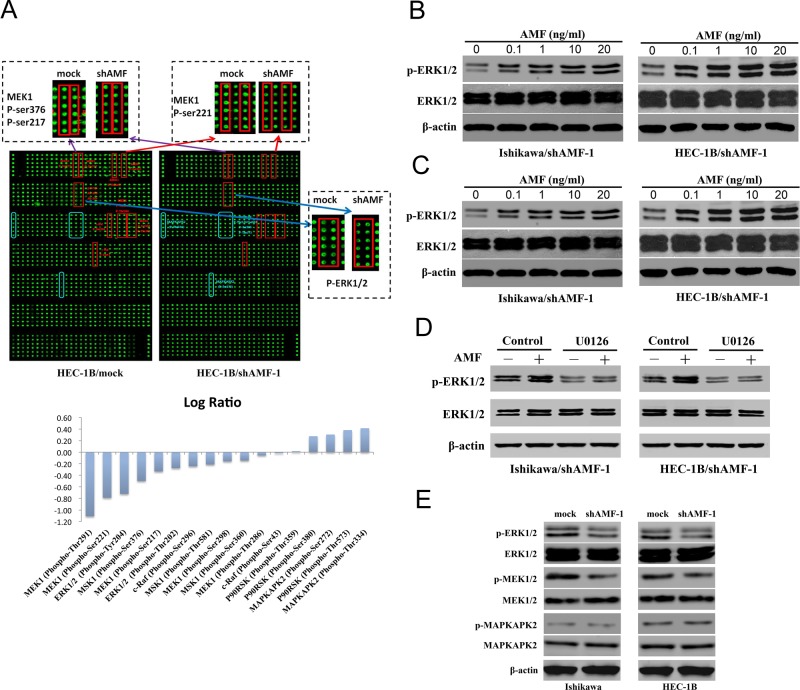
MAPK signaling pathway is involved in AMF/PGI-mediated activation **A.** The anti-phosphoprotein microarray identified protein factors whose phosphorylation states changed in HEC-1B cells when AMF was stably knocked down by shRNA (Top). The signal intensities of phosphorylated proteins and total protein levels were determined. The log-fold changed of each protein was determined as the ratio between the percentages of phosphorylated proteins in total proteins in HEC-1B/mock and HEC-1B/shAMF-1 (Bottom). In the top panel, the red squares show the downregulation of the phosphorylated proteins, and blue squares show upregulation of the phosphorylated proteins between HEC-1B/mock and HEC-1B/shAMF-1 cells. **B.** and **C.** Ishikawa and HEC-1B cells were serum-starved for 16 hours were stimulated with purified PGI; p-ERK levels were monitored by western blot for dose-dependent effects of PGI (0–20 ng/mL) after 15 minutes (B) and for time-dependent dffects (0–30 min) with 10 ng/mL PGI (C). **D.** The effect of U0126 (20 μmol/L; MAPK inhibitor) on PGI-induced ERK1/2 phosphorylation assessed by western blotting. Serum-starved cells were pretreated with U0126 for 1 hour before PGI (10 ng/mL) stimulation for 15 minutes. **E.** The effect of AMF knockdown on ERK1/2, MEK1/2, and MAPKAPK2 phosphorylation was evaluated by western blotting; β-actin was used as a loading controls.

The tumorigenic activation by AMF/PGI is mediated by its interaction with receptors on the surface of target cells [15, 33], such as AMFR/gp78, a G-protein–coupled receptor (GPCR) family number [14]. Receptors stimulated with AMF/PGI leading to the activation of signaling pathways are relevant to G protein mediated signaling pathways including MAPK/ERK axis [19], AKT pathway [18, 34] and mTOR pathway [35]. To identify the pathways that are involved in EC signal transduction of AMF/PGI activation, we used an antibody microarray to detect proteins deactivated by GPCRs under AMF-silencing conditions. A signal intensity ratio of > 1.5 (log-fold change 0.4) or < 0.67 (log-fold change −0.4) indicates a significant change in protein expression. The antibody array showed that silencing AMF in HEC-1B cells (HEC-1B/shAMF-1) markedly inhibited phosphorylation of MAPK pathway proteins MEK1 (at Thr291/Ser221) and ERK1/2 (at Thr202/Tyr204) compared with that of the control cells, whereas phosphorylation of MAPKAPK2 was increased (Figure 4A). To confirm the results, Western blot analyses were performed to assess ERK1/2, MEK1/2, and MAPKAPK2 phosphorylation in shAMF- and mock-transfected Ishikawa and HEC-1B cells (Figure 4B). To further identify whether the MAPK/ERK1/2 pathway is involved in AMF/PGI mediated signaling, the effect of exogenous PGI rescue was studied. The results revealed that purified PGI restored MAPK/ERK1/2 pathway activation in Ishikawa/shAMF-1, Ishikawa/shAMF-2, HEC-1B/shAMF-1 and HEC-1B/shAMF-2 cells (Figure 4C, 4D), and pretreatment with the inhibitor of MAPK (U0126) markedly decreased the phosphorylation of ERK1/2 (Figure 4E). These results were supported by the antibody array results and indicated that MAPK-ERK1/2 pathway activation is involved in AMF/PGI signaling.

### AMF/PGI dual function mediates cell proliferation, migration and invasion activities through MAPK-ERK1/2 signaling

**Figure 5:**
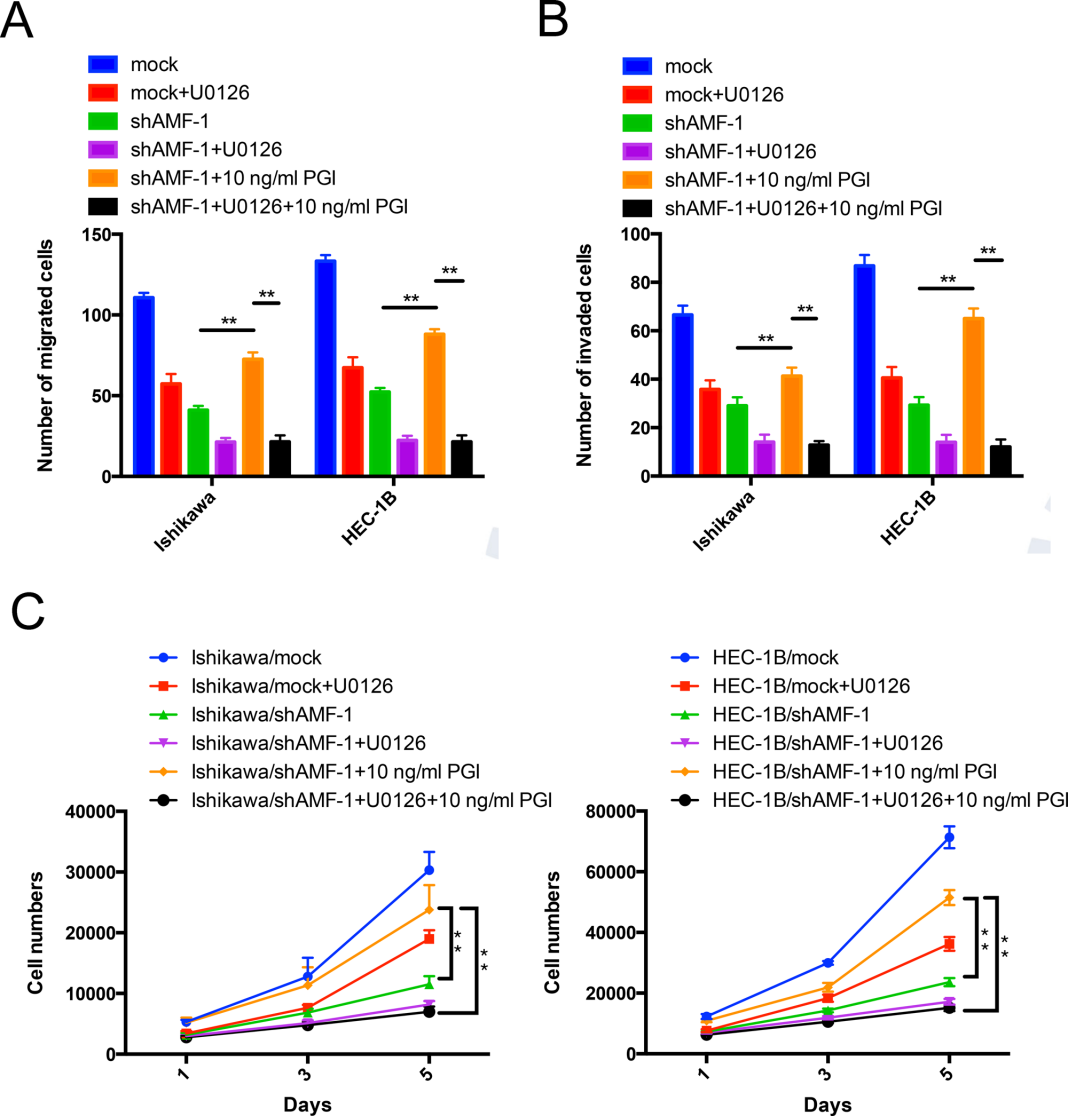
AMF/PGI mediates cell proliferation, migration and invasion activities through MAPK-ERK1/2 signaling **A.** and **B.** The effect of PGI on U0126-inhibited cells migration and invasion. Ishikawa/shAMF-1, HEC-1B/shAMF-1 cells and their mock controls were pretreated with U0126 or DMSO (control) for 24 hours, and cells that had migrated (left) or invaded (right) were counted after 16 h (left) or 24 h (right) in Transwell assays. Quantitative data from 3 different experiments are presented. **C.** The effect of PGI on U0126-inhibited cell proliferation. Cells treated as described above were then counted after 1 day, 3 days, and 5 days using MTS assays. Data represent the mean ± S.D. of the three independent experiments (**P < 0.01).

Previous studies have shown that AMF expression is crucial for cell proliferation, migration and invasion. To further examine whether the effects of PGI function is required to stimulate MAPK-ERK1/2 activity in EC cells, shAMF- and mock-transfected Ishikawa and HEC-1B cells were pre-incubated with U0126 with or without purified PGI and then subjected to motility, invasion and proliferation assays. Pre-incubation of U0126 abolished the PGI-induced increase in cell migration, invasion and proliferation, indicating that activation of MAPK-ERK1/2 was essential for PGI-induced EC cell proliferation, migration and invasion (Figure 5A, 5B and 5C).

### AMF promotes tumor metastasis and proliferation *in vivo*

**Figure 6:**
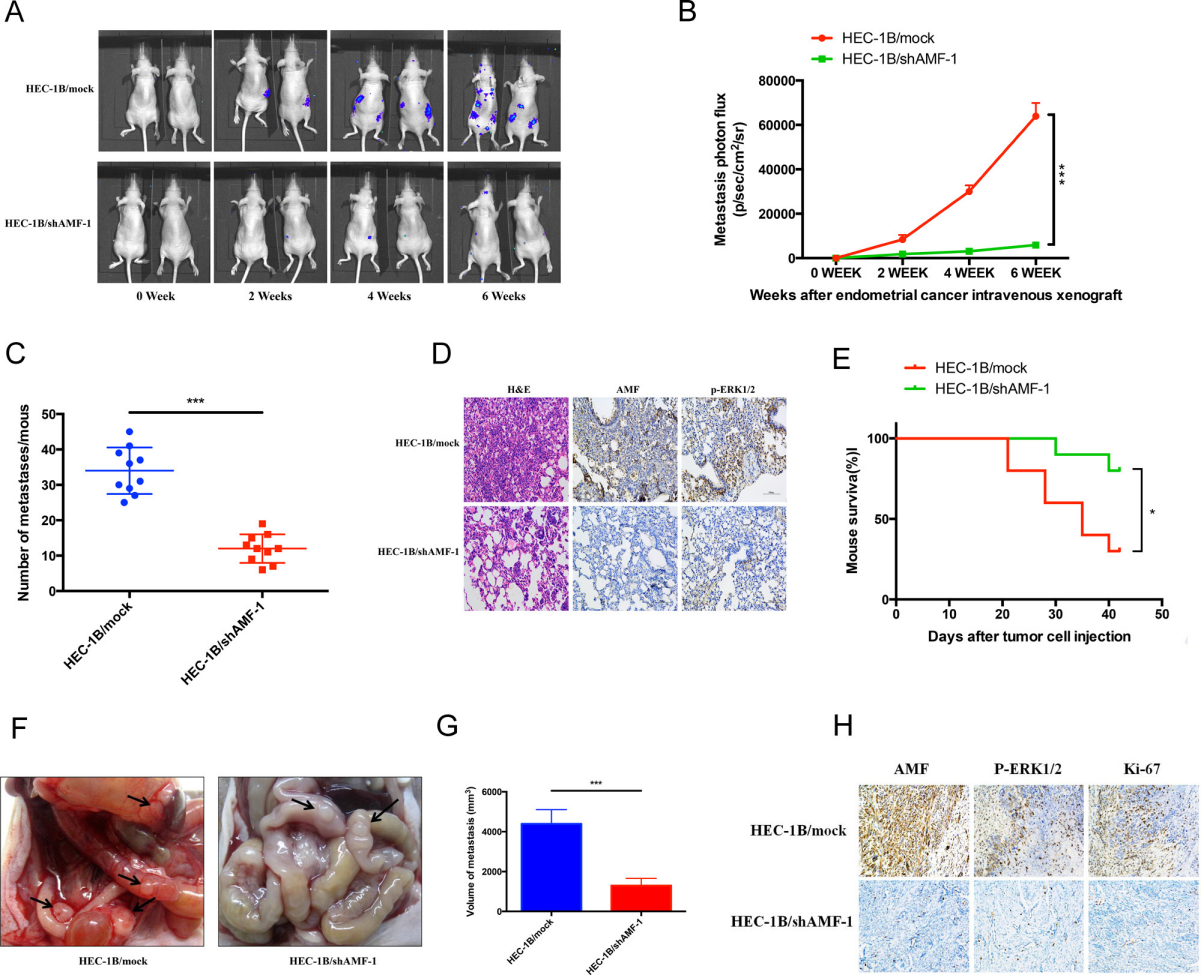
The effects of silencing AMF on development and progression of xenograft tumor formation Five-week-old nu/nu female mice were randomly allotted to two groups (*n* = 10 mice per group) and HEC-1B/mock or HEC-1B/shAMF-1 EC cells were injected with luciferase-labeling (1 × 10^5^ cells per mouse) through the left ventricle of the heart. **A.** Tumor metastasis over a 6-weeks period by bioluminescence analysis. **B.** Quantitative analysis of metastatic cells in lung bioluminescence analysis. The means and 95% confidence intervals (error bars) are presented; ***P < 0.001. *P* values were calculated using a two- sided Student's *t* test. p/sec/cm^2^/sr = photons/second/cm^2^/steradian. **C.** The tumor metastasis per mouse was counted and measured from the mice injected with HEC-1B/shAMF-1 or vehicle control. *P* values were calculated using a two-sided Student's *t* test; ***P < 0.001. **D.** Representative H&E staining histopathology analyses of HEC-1B/mock and HEC-1B/shAMF-1 EC cells lung metastases in mice (left panels). AMF and p-ERK1/2 expression was detected by immunohistochemistry (middle and right panels) (magnification, 200×). **E.** Kaplan–Meier analysis of mouse survival after xenograft. *P* values were calculated using two-sided log-rank test (*, *P* < 0.05). **F.** Macroscopic of intraperitoneal injection tumor metastasis. Black arrow, tumor metastasis. **G.** Average volume of tumor metastasis in the HEC-1B/shAMF-1 and HEC-1B/mock group. *P* values were calculated using a two-sided Student's *t* test; ***P < 0.001. **H.** AMF, p-ERK1/2 and Ki-67 expression was detected by immunohistochemistry (magnification, 200×) in intraperitoneal injection tumor metastasis model.

To evaluate the potential of AMF as a carcinogenic factor in EC, we next investigated whether AMF silencing decreased endometrial cancer progress in tumor metastasis and growth model. HEC-1B/mock control and HEC-1B/shAMF-1 transfected with luciferase clones (Supplementary Figure S1) were inoculated into nude mice via left ventricular or intraperitoneally, The mice were monitored for 42 days. During this period, tumor growth was serially observed by bioluminescence imaging. Tumor metastasis was dramatically reduced in mice injected with HEC-1B/shAMF-1 cells compared to mice injected with control cells (HEC-1B/mock) (Figure 6A and 6B). To confirm this observation, pathologic anatomy analysis of the numbers of metastasis were performed (*p* < 0.001, Figures 6C). Our results indicated that stable knockdown of AMF substantially abrogated the growth of HEC-1B EC cells. To verify its effects, tumor tissues were examined with hematoxylin-eosin (H&E) and immunohistochemistry; AMF and phospho-ERK1/2 levels were lower in the HEC-1B/shAMF-1 tumors than in control tissues (Figure 6D), suggesting that AMF highly inhibited EC cell metastasis via MAPK-ERK1/2 pathway *in vivo*. As a result, survival of shAMF-treated tumor-bearing mice was prolonged significantly (Figure 6E).

To further prove our results, peritoneal disseminated tumor growth assays in nude mice were performed. As shown in Figure 6F, tumor growth was significantly abrogated in the HEC-1B/shAMF group. The volume of AMF silenced tumors was significantly smaller than the control group (Figure 6G). Immunohistochemical analysis showed that the expression of AMF, phospho-ERK1/2 and Ki-67 was decreased in the HEC-1B/shAMF tumors, which confirmed our previous results *in vitro* and revealed that AMF restrained the growth and metastasis of EC cells *in vivo* (Figure 6H).

## DISCUSSION

Even though most endometrial carcinomas are diagnosed at an early stage, there is still a 15–20% chance of EC reoccurrence and systemic therapies in metastatic disease are of limited efficacy [36]. Cancer metastasis is a multistep process involving complex and highly coordinated interactions between tumor cells and a constantly changing host microenvironment [37]. EC resulting from the accumulation of acquired genetic mutations has been well characterized, although the molecular mechanisms linking the genetic changes to the aggressive nature of this disease remain poorly understood. It has been long recognized that AMF/PGI is oncogenic in tumors of neurogenic origin, such as neuroblastoma [38]. Furthermore, recent work has shown that its oncogenic role in other tumors, including its ability to regulate aggressive tumors, is quite consistent with its role in neurogenic diseases [14, 36, 39]. In this study, for the first time, we found that AMF/PGI played an important role in EC aggressiveness through the MAPK-ERK1/2 signaling pathway. The most interesting result was the significant increase in AMF levels in the serum of EC patients, supporting previous reports of AMF as a serum cancer marker.

It has been reported that AMF/PGI expression is induced in various types of cancer compared with healthy tissues, and increases with malignancy [20, 40]. The function of AMF/PGI in EC has not yet been reported.. In our study, we demonstrated that AMF was aberrantly expressed in EC tissue compared to normal epithelium, which was consistent with a previous study showing that AMF is a tumor-secreted polypeptide identified by its ability to induce the migration of the producing cells [41]. In the case of AMFR, there were different views about its distribution and localization in cells. Watanabe, H. et al. believed that AMFR/gp78 was expressed on the cell surface, which made sense because of its binding function with AMF [42]. Another view demonstrated that AMFR/gp78 was mainly distributed in the smooth endoplasmic reticulum, functioning as an E3 ubiquitin ligase [43]. Therefore, the precise positioning of AMFR/gp78 needs further confirmation.

PGI is an example of a ‘moonlighting’ protein that exhibits multiple cellular functions, including acting as a secreted cytokine or glycolytic enzyme [44]. To distinguish AMF impacted cell migration and invasion via functions of its cell surface cytokine function or potential impacts on glycolysis, we measured intracellular PGI activity and found that silencing of AMF by shRNA did not affect the enzymatic activity of PGI in both EC cell lines. Previously, it was reported that the cytokine function of PGI has evolved during evolution independently of the enzymatic activity [44]. There was also experimental evidence that a dichotomy exists between the enzymatic and cytokine activities [45]. The shRNA designed to target AMF was a very efficient suppressor of expression. In our study, we observed decreased levels of AMF secretion but no affect on enzymatic activity, which was consistent with the above experimental results, though the mechanism needs further investigation. AMF knockdown suppressed EC cell migration, invasion and proliferation, indicating that AMF promotes EC progression in an autocrine manner.

During tumor formation and metastasis, AMF/PGI may exert its anti-apoptotic abilities [46], cause acceleration of cell cycle transition [47] and cell transformation [31], and induce tumor cell migration and invasion [48]. Efforts have been made to elucidate the mechanisms that mediate the effects of AMF, and they revealed that several signaling pathways and molecules were involved, for example, small GTPase RhoA followed by JNK [28, 47], RhoC [49], MEK1/2 phosphorylation [47], protein kinase C [46], and PI3-Akt pathways [18, 32]. In an attempt to shed light on the complex mechanisms involved in specific AMF/PGI functions, we used shAMF- and mock-transfected HEC-1B endometrial cells in an anti-phosphoprotein array to identify a unique AMF–induced ‘molecular signature’ in the development of cell growth and motility. The analysis established a pathway involving MAPK-ERK1/2 signal transduction, which promotes and induces cell migration. This signaling pathway has been proposed in other systems, some of which apply to cancer cell invasion, migration and metastasis [19, 50]. However, when shAMF transfectants were exposed to exogenous PGI and pretreated with U0126, phospho-ERK1/2 levels were reduced, indicating the role of MAPK-ERK1/2 activation in AMF/PGI signaling. Another interesting finding of this study is that exposing AMF-silenced EC cells to PGI restored the migration, invasion, and proliferation, and inhibition of MAPK-ERK1/2 pathway. In contrast, using U0126 counteracted those effects of exogenous PGI on EC cells, suggesting that both AMF and PGI functions are mediated by the MAPK-ERK1/2 pathway in EC cells and thus exhibit this effect on EC progress.

AMF/PGI promotes the malignancy of several cancers and silencing AMF may present an approach to the prevention or treatment of malignant tumors *in vivo*. Thus far, only very few studies about silencing of AMF *in vivo* have been described [16]. Further investigation is required to identify effective therapy strategies using AMF shRNA approaches, given the important role of AMF in cancer tissue. Alternatively, small-molecule antagonists against AMF/PGI may be useful as anti-tumor or metastasis agents [51, 52].

In this study, multiple methods have been applied to profile the important roles of AMF/PGI involvement in EC. However, there are still a few limitations regarding clinic research. For example, even though the expression of AMF in endometrial tissue was detected, along with AMF/PGI in the serum of EC patients, the correlation between the clinicopathologic characteristics of EC patients and the expression of AMF in EC endometrial tissue and serum should be identified. Further more, the relationship between AMF/PGI expression and clinical EC pathology should be investigated to determine whether AMF/PGI has the potential as a biomarker to predict EC disease prognosis.

Our results show that AMF was highly expressed in human EC cell lines and endometrial tissue in EC patients. AMF knockdown reduced the growth, invasion, and migration of the EC cells both *in vitro* and *in vivo*. Exogenous PGI stimulation induces the promotion of EC cell migration, invasion, and proliferation, likely through MAPK-ERK1/2 signaling pathway, which suggests that AMF/PGI plays important roles in EC occurrence and progression. AMF/PGI might be a potential therapeutic target in EC and *in vitro* and *in vivo* studies with AMF/PGI antagonists should be implemented in the near future.

## MATERIALS AND METHODS

### Reagents and antibodies

Purified rabbit PGI was purchased from Sigma for exogenous PGI stimulation (cat. NoP9544). Mouse monoclonal anti-AMF (ab66340), rabbit polyclonal anti-AMF (ab86950), and anti-β-actin for use in immunohistochemistry or western blot analyses were obtained from Abcam Ltd. (Hong Kong, PR China). Antibody against p-MEK1/2, MEK1/2, p-ERK1/2, ERK1/2, p-MAPKAPK2 and MAPKAPK2 were from Cell Signaling Technology (Shanghai, PR China). U0126 (MAPK inhibitor) was purchased from Selleck.cn.

### Patients and tissue samples

For this study, tissues from 72 cases of endometrial cancer and 32 cases of normal endometrium were obtained from surgical procedures performed in International Peace Maternity & Child Health Hospital between 2010 and 2013. This project was approved by Human Investigation Ethics Committee of the International Peace Maternity and Child Health Hospital, and the informed consent was obtained from all patients before the study.

### Immunohistochemistry and ELISA

Paraffin-embedded EC and normal endometrium tissue sections (4 μm) were processed for immunohistochemistry. Briefly, after deparaffinization and dehydration, specimens were incubated with a specific polyclonal antibody raised against purified rabbit AMF (ab86950), at 1:200 dilution. Antibody binding was detected using Envision reagents (Boster bioengineering, Wuhan, China) according to the manufacturer's instructions. For evaluation of AMF expression, staining intensity was scored as 0 (negative), 1 (weak), 2 (medium), or 3(strong). The extent of staining was scored as 0 (0%), 1 (1%–25%), 2 (26%–50%), 3 (51%–75%), or 4 (76%–100%), according to the percentage of the positively stained areas in relation to the whole tumor area. The sum of the intensity score and the extent score was used as the final staining score (0–7). Tumors with a final staining score of 4 or higher were considered “positive” [53]. The results were assessed by two pathologists who were blind with respect to the tissue source. To measure serum AMF level, serum were collected from 15 normal women and 15 EC patients and subjected to ELISA using a commercial human AMF kit (Abcam, ab171575).

### Cell culture

Ishikawa and HEC-1B cells were purchased from the American Type Culture Collection (Manassas, VA) and maintained according to the provider's instruction in DMEM/F12 media (Gibco, Auckland, NZ) supplemented with 10% FBS (Gibco, Carlsbad, CA). All cells were grown until confluent and were incubated in serum-free medium for 24 h before treatment with various experimental agents.

### Stable silencing of AMF expression by short hairpin RNA (shRNA)

AMF shRNA constructs were cloned into pLKO.1 plasmid under the control of U6 promoter for stable expression (Sigma) [54]. Three pairs of annealed DNA oligonucleotides were inserted into the AgeI and EcoRI restriction sites of pLKO.1. The most effective pairs of sequence targeted to human AMF is: sense 5′- CGCCATGTATGAGCACAAGAT-3′ and anti-sense 5′- GCGGTACATACTCGTGTTCTA-3′ (shAMF-1), as well as sense 5′-CCTGTCTACTAACACAACCAA-3′ and anti-sense 5′-GGACAGATGATTGTGTTGGT T-3′ (shAMF-2), respectively. Ishikawa and HEC-1B cells were transfected with lentiviral vector pLKO.1 (mock) or AMF-specific shRNA lentiviral particles in six-well plates in the presence of polybrene (6 mg/mL) and then treated with puromycin (2 mg/mL) to generate stable clones. Puromycin-resistant AMF knockdown clones were harvested by ring selection, and AMF gene expression and protein level were confirmed by qRT-PCR and immuneblotting.

### RNA extraction and qRT-PCR (quantificational real-time polymerase chain reaction)

Total RNA was isolated using Trizol reagent (Invitrogen, Life Technologies; Shanghai, PR China) and reverse transcribed using a reverse transcriptase kit (Takara, Dalian, PR China). Gene expression was detected with SYBR green master mix (Takara, Dalian, PR China) on an ABI Prism 700 thermal cycler (Applied Biosystems, Foster City, CA, USA). Gene expression was calculated using the 2^(−ΔΔCt)^ formula and normalized against β-actin. The oligonucleotide primers were 5′-CGCCCAACCAACTCTATTG-3′ (forward) and 5′-GATGATGCCCTGAACGAAG-3′ (reverse) for human AMF detection; 5′-CAGCC ATGTACGTTGCTATCCAGG-3′ (forward) and 5′-AGG TCCAGACGCAGGATGGCATG-3′ (reverse) for human β-actin detection (as a housekeeping gene). All experiments were performed independently in triplicate.

### Protein extraction and western blot analysis

For whole-cell lysates, cells were washed twice with PBS and collected by scraping. Cell pellets were lysed in cold precipitation assay buffer [20 mM Tris-HCl (pH 7.4), 150 mM NaCl, 10mM EDTA, 1% NP40, Triton X-100, sodium deoxycholate] containing 1 mmol/L DTT, 1 mmol/L phenylmethylsulfonyl fluoride, 10 μg /mL leupeptin, and 10 μg/mL aprotinin. Samples were separated by centrifugation (15,000 rpm in 4°C for 30 min). Lysate supernatants were 100-fold concentrated with Amicon Ultra (30,000 nominal molecular weight limit; Millipore).

The extracted protein concentration was measure with BCA Protein Assay Kit (Thermo Scientific). Equal amounts of the proteins (20 μg of cellular protein or 80 μg of secreted protein) were separated on 10% SDS-PAGE gels and transferred to 0.2-Am PVDF membrane (Osmonics, Inc.). Each Membranes was then incubated overnight at 4°C with an appropriate diluted primary antibodies. HRP-secondary anti-rabbit or anti-mouse antibodies (diluted 1:5000 to yield 0.2 mg/mL; Abcam) were used to detect the bound primary antibodies. Blots were visualized using the Bioscience Odyssey Infrared Imaging System (LI-COR Biosciences), and band density was quantitated using the Image-J imaging analysis software (NIH, Bethesda, MD). Data were normalized to β-actin expression by densitometry, and statistical data from at least three experiments was graphed.

### PGI enzymatic activity

The enzymatic activities of PGI proteins were measured as described previously [55]. Briefly, 48 h after transfection with vector and shAMF/PGI, cells were lysed in radioimmune precipitation assay buffer, and each sample at 30 μg/protein was used to measure their enzymatic activities at A340 nm using a Shimadzu spectrophotometer.

### AMF secretion in conditioned media

Confluent cells were cultured for 3 days in completed medium, 10% FBS, and then were starved with serum-free medium for 24 h. The supernatant was filtered through a 30-kDa cutoff filter (Millipore), and AMF secretions were measured by Western blot analysis.

### Transwell assay

A total of 1 × 105 cells in serum-free medium were introduced into the upper compartment of Transwell cell culture chambers (8 μm pore size; Corning Costar No. 3422) with or without Matrigel-coating (BD Biosciences). The bottom chamber was filled with PGI (10 μg/mL), U0126 (20 μmol/L) or DMSO. After 16 h (migration assay) or 24 h incubation (invasion assay which with Matrigel-coating), cells were stained with Calcein-AM (0.2 μg/mL; Invitrogen, NO.C3100MP) for 30 min. The number of cells that had migrated or invaded was counted using MetaMorph image analysis software (Molecular Devices, Sunnyvale, CA), and the result was calculated as mean average ± SD (*n* = 3).

### Cell proliferation assay

For cell proliferation assay, 2 × 10^3^ cells/well were plated in triplicate for each group on 96-well plates, and the media including exogenous PGI, U0126, or DMSO were added the day after plating. Cells were grown for 5 days and fresh medium was provided every day. The number of cells was determined using the MTS kit (Promega, NO.G3580). The metabolically active cells reduced MTS to a soluble formazan product, and the absorbance was measured at 490 nm in a plate reader (Bio-Rad). Wells containing known cell numbers (0, 1000, 2000, 5000, 10000, 20000 or 40000 cells/well; 6 wells/cell density) were treated in the similar fashion to establish standard curves. Three independent experiments were run for the cell proliferation assay.

### Flow cytometry analysis

Whole-cell suspensions were stained with 50 ug/mL PI (propidium iodide) after 70% ethanol fixation and analyzed for cell cycle phase distribution with a BD Biosciences flow cytometer.

### Three-dimensional spheroid culture

Three-Dimensional spheroid culture and analysis was done as described previously with slight modification [56]. The AlgiMatrix 3D Culture System (Invitrogen) was used to construct an artificial bioscaffold to facilitate the three-dimensions spheroid formation in AMF knockdown cells (Ishikawa/shAMF-1 and HEC-1B/shAMF-1) and controls (Ishikawa/mock and HEC-1B/mock), respectively. Cells were seeded into AlgiMatrix six-well plates and grown to form spheroids for 2 weeks and then photographed under a microscope. CellProfiler 2.0 software (http://www.cellprofiler.org/) was used for image processing to detect and quantify individual spheroids.

### Luciferase assay

HEC-1B cells stably transfected with vehicle or shAMF-1 were plated at 1 × 10^5^ cells in six-well plates and grown for 24 hour, and then co-transfected with 200 ng luciferase reporters using Lipofectamine 2000. At two days post-transfection, luciferase and Renilla activity of lysates was measured with Dual-Glo Luciferase Reagents (Promega, E1531). Luciferase activities were normalized against Renilla activity, and relative ratios for each transfection were calculated. Experiments were performed on at least three independent occasions, and error bars indicate SD.

### Phospho-protein profiling using an anti-phosphoprotein array

The anti-phosphoprotein microarray PGP193, which was designed and manufactured by Full Moon Biosystems, Inc. (Sunnyvale, CA), contains 193 antibodies. Each of the antibodies has six replicates that are printed on coated glass microscope slides, along with multiple positive and negative controls. In brief, cell lysates obtained from HEC-1B/mock and HEC-1B/shAMF-1 were biotinylated with the Antibody Array Assay Kit (Full Moon Biosystems, Inc.). The antibody microarray slides were first blocked in a blocking solution (Full Moon Biosystems, Inc.) and dried with compressed nitrogen. The slides were then incubated with the biotin-labeled cell lysates (∼100 μg protein) in coupling solution at room temperature for 2 h and rinsed extensively with Milli-Q grade water before detection of bound biotinylated proteins using Cy3-conjugated streptavidin. The slides were scanned on a GenePix 4000 scanner and the images were analyzed with GenePix Pro 6.0 (Molecular Devices, Sunnyvale, CA). The fluorescence signal for each antibody was obtained from the fluorescence intensity of this antibody spot. A ratio computation was used to assess the extent of protein phosphorylation. For each antibody that has phosphorylated and matching unphophorylated values in both the control data and experiment data are represented as “phospho” and “unphospho”.

### *In vivo* experiments and analyses

The left ventricle of the heart or intraperitoneal injection tumor metastasis model was used to evaluate the effect of silencing AMF on tumor progressive [57, 58]. Twenty 5–6 week-old female athymic nude mice (BALB/c) were obtained from Sino-British Sippr/BK Lab Animal Co, Ltd (Shanghai, China). The mice were randomly divided into two groups (*n* = 10 mice in each group). All mouse studies were performed in accordance with animal protocol procedures approved by the Department of Laboratory Animal Science at the School of Medicine, Shanghai Jiao Tong University. Excess anesthetic was used to euthanize mice at the end of the experiment.

For this protocol, AMF stable knockdown cells (HEC-1B/shAMF-1) and the control cells (HEC-1B/mock) stably expressing firefly luciferase were resuspended in sterile PBS (50 μl) and injected (5 × 104 cells/injection) into the left ventricle of the heart or intraperitoneal of 8-week-old female BALB/c mice. Metastasis was determined at 3, 14, 21 and 28 days post-injection by bioluminescent imaging on a Xenogen IVIS-200 (Caliper Life Sciences, Hopkinton, MA). At day 42, the mouse were injected with control cells (HEC-1B/mock) were all nature dead meanwhile the mice left were killed, and then any disseminated tumors of >1 mm in diameter were resected and counted for each mouse. The volume of the tumor metastasis was measured by the sum of all disseminated tumors, and the tumor volume was calculated as (Rmax) × (R2 - min)/2. HEC-1B/mock and HEC-1B/shAMF-1 tumor metastases were counted, and serial histologic sections of tumor metastasis were processed for H&E staining and histological examination. Procedures for IHC analysis of AMF, p-ERK1/2 and Ki-67 were done as described above.

### Statistical analysis

Continuous variables were recorded as mean ± SD and all statistical analyses were done using Statistical Package for the Social Sciences (SPSS) software version 17.0 (Chicago, IL, USA). Volumetric data were assessed using an unpaired Student's *t* test, or one-way ANOVA analysis followed by post-hoc LSD test or Dunnett's test for multiple comparisons. Survival curves were assessed using standard log-rank test and by the Kaplan-Meier method. *P* values < 0.05 were considered statistically significant. All experiments were repeated independently at least three times.

## SUPPLEMENTARY FIGURE


