# Correction: AMF/PGI-mediated tumorigenesis through MAPK-ERK signaling in endometrial carcinoma

**DOI:** 10.18632/oncotarget.27359

**Published:** 2020-02-18

**Authors:** Yiran Li, Yuanhui Jia, Qi Che, Qian Zhou, Kai Wang, Xiao-Ping Wan

**Affiliations:** ^1^ Department of Obstetrics and Gynecology, Shanghai First People’s Hospital Affiliated to Shanghai Jiao Tong University, Shanghai, China; ^2^ Clinical and Translational Research Center, Shanghai First Maternity and Infant Hospital, Tongji University School of Medicine, Shanghai, China; ^3^ Department of Gynecology, Shanghai First Maternity and Infant Hospital, Tongji University School of Medicine, Shanghai, China

**This article has been corrected:** For purposes of clarity, Figure 2B has been updated. In the corrected Figure 2B, a clear vertical line was added to each band, as well as two new labels (“exogenous synthetic” and “cell component”) in the bottom of the figure in order to more clearly point out that the left side of the line represents exogenous synthetic AMF, while the right side is cell component. In addition, the figure legend of Figure 2B has been corrected to read: “B. Top, Immunoblot analysis for AMF protein and β-actin expression. The band on the left side of the vertical line were exogenous synthetic, and the band on the right side were cell component. Bottom, quantification analysis of AMF expression. AMF cytokine itself was used as positive control.”

There is also a correction to Figure 4. During figure assembly, the Figure 4B was pasted twice (classified by concentration) and the Figure 4C (classified by time) was omitted. The corrected Figure 4, obtained using original data, is shown below. The authors declare that these corrections do not change the results or conclusions of this paper.

Original article: Oncotarget. 2015; 6:26373–26387. 26373-26387. https://doi.org/10.18632/oncotarget.4708

**Figure 2:**
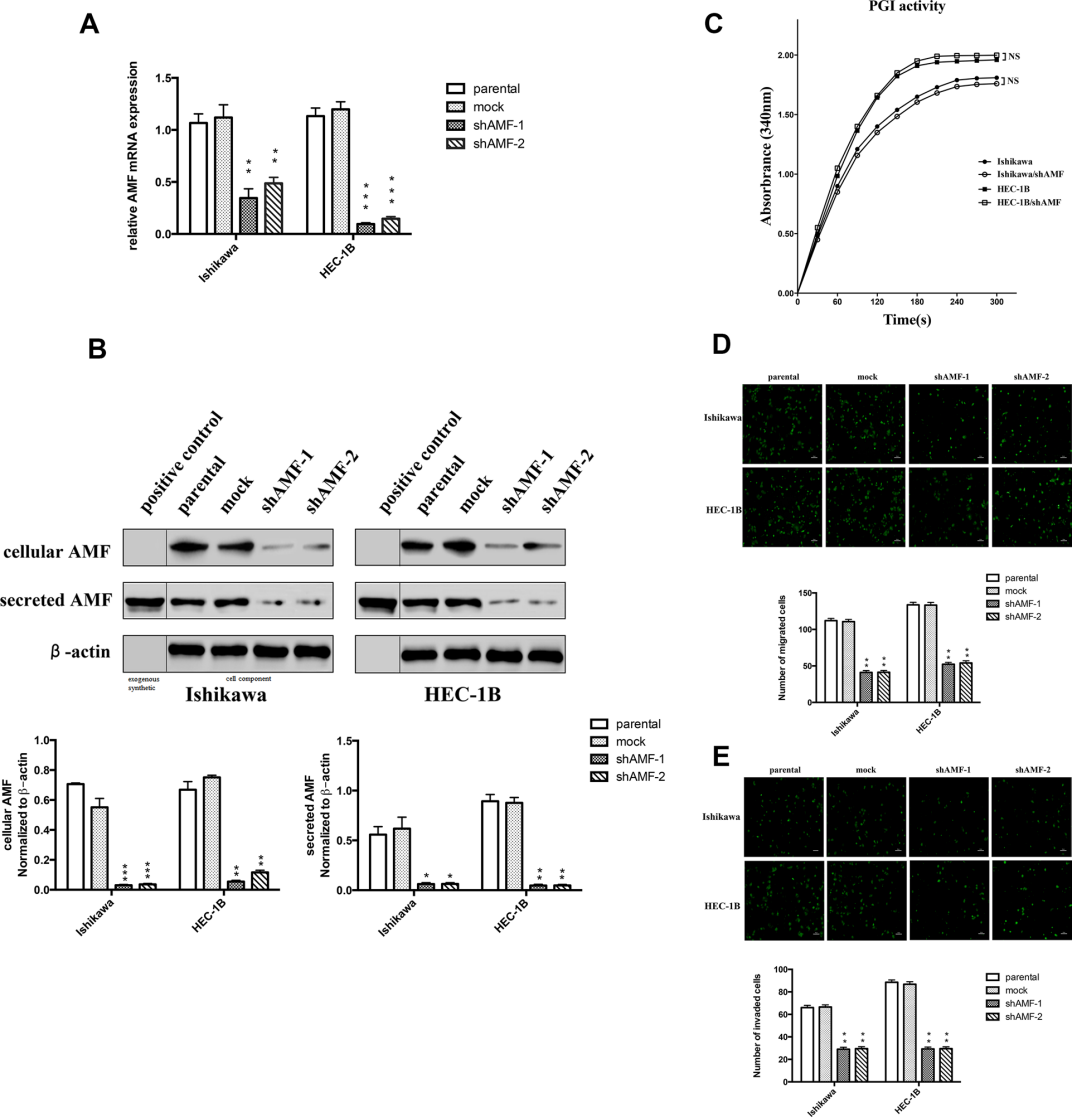
Effect of AMF gene silencing on EC cells migration and invasion.

(**A**) Ishikawa and HEC-1B cells were stably transfected with plasmid containing AMF-specific shRNA (shAMF-1 or shAMF-2)) or control plasmid (mock). Cells were then analyzed by qRT-PCR. (**B**) Top, Immunoblot analysis for AMF protein and β-actin expression; Bottom, quantification analysis of AMF expression. AMF cytokine itself was used as positive control. (**C**) Intracellular PGI/AMF enzyme activity was measured in EC cell lines by enzymatic spectrophotometer assay. (**D** and **E**) Transwell assay for cells migration (**D**) and invasion (**E**) Cells were seeded onto the upper surface of Transwell chambers without (**D**) or with Matrigel-coated (**E**) After 16 h (**D**) or 24 h (E) of incubation, the penetrating cells were stained with calcein-AM and recorded under a microscope. Top, Photographs depict the migration or invasion of EC cells; Bottom, quantitative data are expressed as mean average ± SD from three independent experiments. (*, *P* < 0.05; **, *P* < 0.01, ***P < 0.001, compared with the control cells).

**Figure 4:**
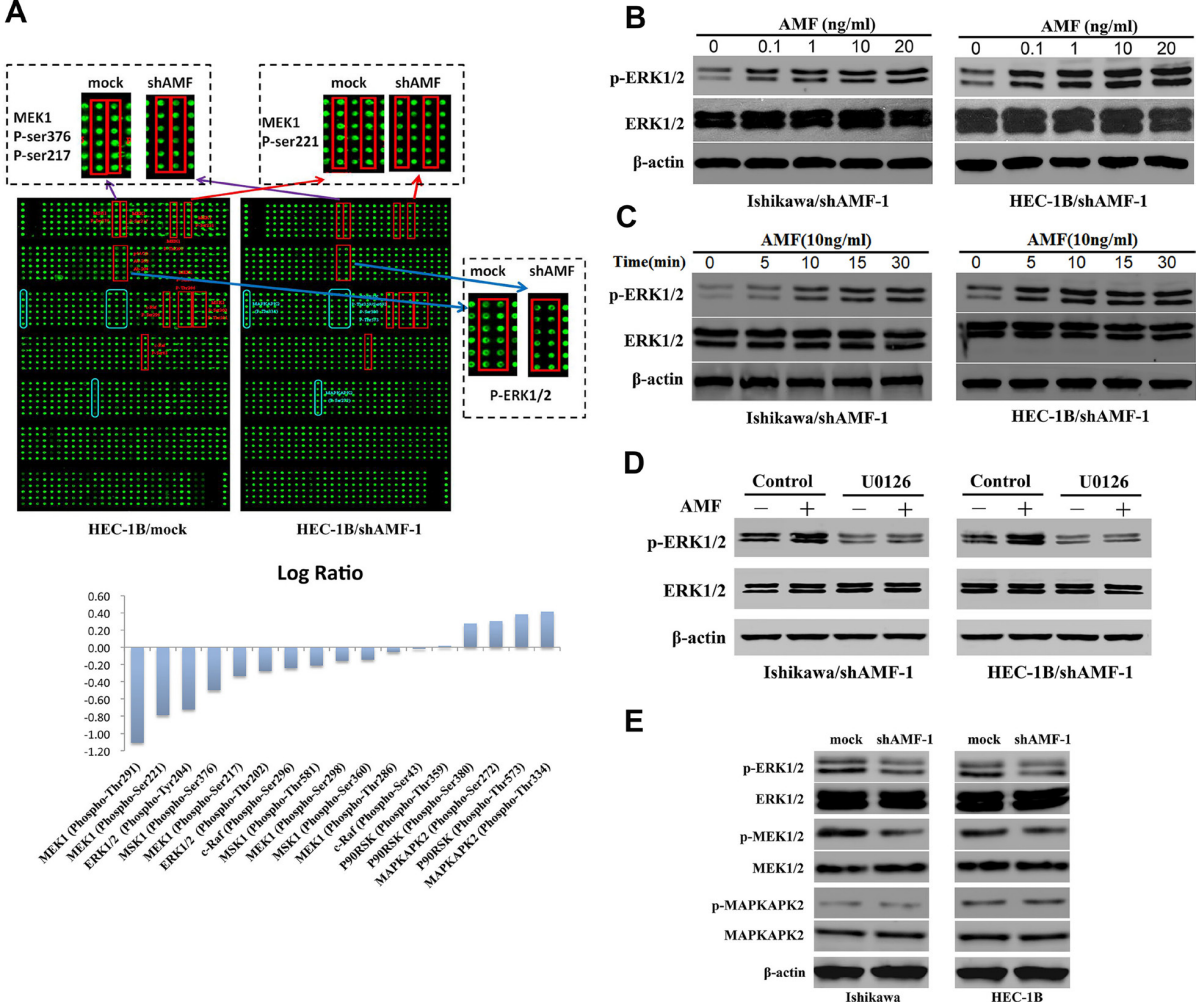
MAPK signaling pathway is involved in AMF/PGI-mediated activation.

(**A**) The anti-phosphoprotein microarray identified protein factors whose phosphorylation states changed in HEC-1B cells when AMF was stably knocked down by shRNA (Top). The signal intensities of phosphorylated proteins and total protein levels were determined. The log-fold changed of each protein was determined as the ratio between the percentages of phosphorylated proteins in total proteins in HEC-1B/mock and HEC-1B/shAMF-1 (Bottom). In the top panel, the red squares show the downregulation of the phosphorylated proteins, and blue squares show upregulation of the phosphorylated proteins between HEC-1B/mock and HEC-1B/shAMF-1 cells. (**B** and **C**) Ishikawa and HEC-1B cells were serumstarved for 16 hours were stimulated with purified PGI; p-ERK levels were monitored by western blot for dose-dependent effects of PGI (0–20 ng/mL) after 15 minutes (B) and for time-dependent dffects (0–30 min) with 10 ng/mL PGI (C). (**D**) The effect of U0126 (20 μmol/L; MAPK inhibitor) on PGI-induced ERK1/2 phosphorylation assessed by western blotting. Serum-starved cells were pretreated with U0126 for 1 hour before PGI (10 ng/mL) stimulation for 15 minutes. (**E**) The effect of AMF knockdown on ERK1/2, MEK1/2, and MAPKAPK2 phosphorylation was evaluated by western blotting; β-actin was used as a loading controls.

