# Supplementary material for: AMF/PGI-mediated tumorigenesis through MAPK-ERK signaling in endometrial carcinoma
Source: Oncotarget. 2015 Jul 20;6(28):26373–87. doi: 10.18632/oncotarget.4708 (PMC4694908; doi:10.18632/oncotarget.4708)
Supplement: Supplementary file 1 [file oncotarget-06-26373-s001.pdf]

SUPPLEMENTARY FIGURE

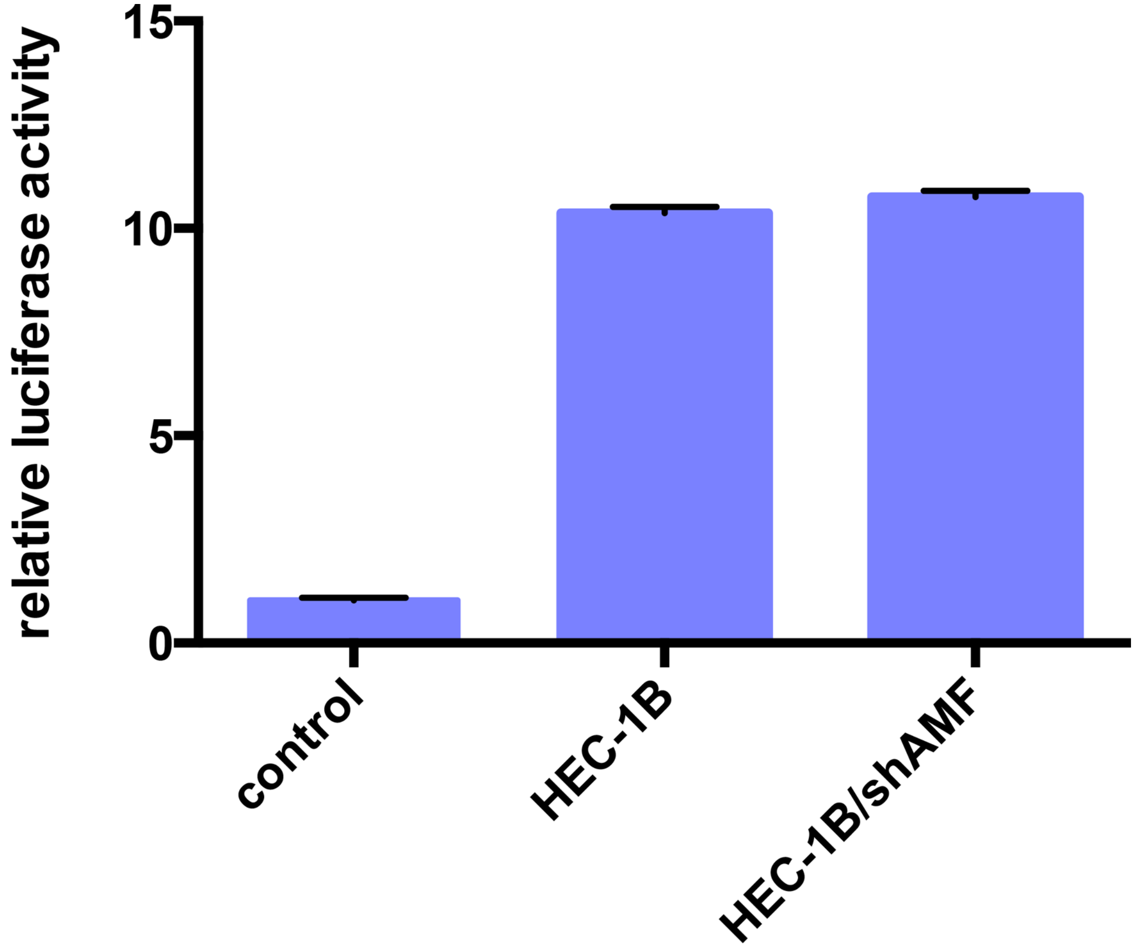

Supplementary Figure S1: Luciferase activity assay in HEC-1B and HEC-1B/shAMF cells.
